# Cyclopentanone Derivative Attenuates Memory Impairment by Inhibiting Amyloid Plaques Formation in the 5xFAD Mice

**DOI:** 10.3390/ijms22179559

**Published:** 2021-09-03

**Authors:** Rahim Ullah, Gowhar Ali, Ajmal Khan, Sajjad Ahmad, Ahmed Al-Harrasi

**Affiliations:** 1 Department of Pharmacy, University of Peshawar, Peshawar 25120, Pakistan; 2 Department of Neurology, Northwestern University Feinberg School of Medicine, Chicago, IL 60611, USA; 3 Natural and Medical Sciences Research Center, University of Nizwa, Birkat-ul-Mouz 616, Nizwa, Oman; ajmalkhan@unizwa.edu.om; 4 Department of Health and Biological Sciences, Abasyn University, Peshawar 25000, Pakistan; sahmad@bs.qau.edu.pk

**Keywords:** cyclopentanone derivative, Alzheimer’s disease, 5xFAD mice, neuroprotection, beta-secretase, beta amyloid plaques, Barnes maze

## Abstract

Alzheimer’s disease (AD) is a chronic neurodegenerative disorder. This study was designed to investigate the effects of cyclopentanone derivative i.e., 2-(hydroxyl-(3-nitrophenyl)methyl)cyclopentanone (3NCP) on behavior, amyloid β (Aβ) plaque deposition, and βAPP cleaving enzyme-1 (BACE-1) expression in the 5xFAD mouse brain. In this study, computational studies were conducted to predict the binding mode of the 3NCP with target sites of the β-secretase. In vivo studies were performed on the 5xFAD mice model of AD using different behavioral test models like light/dark box, elevated plus maze (EPM), and the Barnes maze tests for the assessment of anxiety, spatial learning and memory. The thioflavin-S staining, immunohistochemistry (IHC), and RT-PCR studies were carried out to find the effect of the 3NCP on the β-amyloid plaques formation and BACE-1 expression. The results of the computational studies showed that the 3NCP has excellent binding affinities for beta-secretase. The light/dark box study depicted that the 3NCP does not cause anxiety. The 3NCP treatment effects in the EPM and Barnes maze tests showed a significant effect on learning and memory. Furthermore, the results of the thioflavin staining and IHC revealed that the 3NCP significantly reduced the formation of the beta-amyloid plaques in brain tissues. Moreover, the RT-PCR study showed that 3NCP significantly reduced the BACE-1 expression in the brain. Conclusively, the results of the current study demonstrate that the 3NCP may be a potential candidate for AD treatment in the future.

## 1. Introduction

Alzheimer’s disease (AD) is the most common progressive neurodegenerative disorder and affects memory-related brain areas [1]. Effective treatment of AD will reduce the burden on the health care system of the country, both in terms of cost and health [2]. In the year 1901, the German psychiatrist and neuropathologist Dr. Alois Alzheimer described AD in a 51-year old patient named Auguste Deter who was suffering from chronic dementia and died in 1906. Her brain was analyzed by Alzheimer for the first time and he found extracellular tangles and intracellular plaques [3]. She was suffering from aphasia, memory impairment, hallucinations, psychosocial disorientation, and incompetence. After her death, Alzheimer examined her brain tissues with a silver-staining technique and observed fibrillary bundles (neurofibrillary tangles) and small miliary foci (senile plaques) from such type microscopic analyses [4,5]. AD was officially listed as a disorder in 1970 [4].

Neuritic plaques (senile plaques) are neuropathologic hallmarks of AD. Neuritic plaques mostly accumulate in the extracellular spaces of the hippocampus and these plaques are composed of peptide “Amyloid-beta (Aβ)” [6,7]. Sequences of Aβ of 40 and 42 (Aβ40, Aβ42) are commonly found in AD, with aggregation of Aβ42 into β-pleated sheets, and Aβ40 remaining in a less toxic monomeric peptide sequence. These monomers aggregate with large concentrations and form oligomer. More oligomers cluster together forming Amyloid β-sheets after structural conformational changes [8]. The accumulation of these peptides leads to neurodegeneration [9]. The therapeutic strategies at the systemic level to prevent or reverse the Aβ deposition could lead to partial restoration of the function of the neuronal circuits. Hence, most approaches for AD treatment aim to prevent or reverse the deposition of Aβ plaque [10].

The β-secretase/BACE-1, also called βAPP cleaving enzyme (BACE) found in neurons, is the only enzyme solely responsible for pro-amyloidogenic APP processing [11], and cleaves APP in the extracellular domain leading to the secretion of Aβ as well as the generation of soluble βAPP (βAPPs) and APP intracellular domain (AICD) fragments. A β-carboxy-terminus fragment (β-CTF) is generated in addition to the βAPPs; β-CTF is a toxic species [12]. BACE-1 knockout removes β-CTF generation, Aβ production, and reduces Aβ plaques pathology, therefore BACE-1 is an important target for therapy of AD [13,14,15,16].

The 5xFAD transgenic mice model is a well-known rodent model used in the preclinical study of AD. These mice present the common AD pathologies at an early age. At two months of age, they aggressively form the Amyloid beta plaques, with cognitive impairment occurring at four months of age, and neuronal loss cause at nine months [17,18]. These animals exhibited APP plaques in different brain areas and also increased Aβ1-40 and Aβ1-42 levels in the brain and cerebrospinal fluid. Such animals show distinct neuroinflammation that is often associated with plaques. Increased oxidative stress, a disturbed blood-brain-barrier homeostasis, and cerebral amyloid angiopathy can be appraised in this model as well [19,20].

Current FDA-approved drugs only alleviate the secondary symptoms of AD but do not treat the underlying disease or delay its progression [21].

There is no effective drug in the market to treat AD, hence, research is occurring across the globe to find out therapeutically effective drugs for this disease [22]. Indeed, the real promise of metabolic/ketogenic approaches is that by altering cellular energetics, oxidative stress, and inflammation, ketogenic (and possible 3NCP) have the potential to do more than provide symptomatic relief but also have the potential to alter disease trajectory. The cyclopentanone derivatives including 3NCP that were tested in our lab exhibited anti-cholinesterase, anti-oxidant, and anti-neuroinflammatory potential [23,24]. The 3NCP has a nitrophenyl group, and the literature studies revealed that compounds containing nitrophenyl group have the potential to inhibit *AChE*, *BChE*, MAO-B, and relieve oxidative stress [25]. In fact, the nitrobenzaldehyde derivatives showed significant antioxidant activity due to the nitro group attached to the benzene ring [26,27]. The 3NCP was synthesized by the previously reported method [28,29].

In this regard, the neuroprotective effect of 3NCP in the transgenic 5xFAD mouse model was evaluated along with spatial memory and learning, and anxiety. Moreover, attempts were made to examine the potential mechanisms of the neuroprotective effect of 3NCP. For this purpose, the β-amyloid plaques burden was evaluated in the hippocampus and frontal cortex through thioflavin-S staining and immunohistochemistry. Furthermore, the computational study was performed to predict the binding mechanism of 3NCP with β-secretase. The RT-PCR study was carried out to evaluate the effect of the 3NCP on BACE-1 expression in the hippocampus and frontal cortex.

## 2. Results

### 2.1. Computational Study of 3NCP with β-Secretase

#### 2.1.1. Molecular Docking

**Figure 1:**
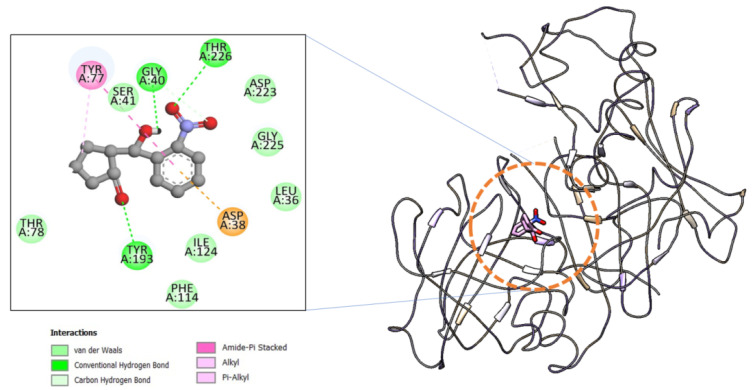
Binding conformation and chemical interactions of the compound at the active pocket of the beta-secretase enzyme.

Molecular docking investigation was performed to determine the binding potential of the 3NCP compound to the beta-site APP cleaving enzyme 1 (BACE-1) in comparison to the co-crystalizedM7D. Additionally, the preferred orientation of the compound was also analyzed and predicted by the docking studies determining how the binding conformation allows stable binding profiles at the binding pocket of the BACE1. The binding energy of 3NCP is −8.1, whereas the control M7D secured binding energy is −8.5 kcal/mol. The compounds are engaged by mixed hydrophobic and hydrophilic interactions which seem key in high complex stability. The highly electronegative oxygen of the compound is involved in close distance hydrogen bonding with Gly40, Tyr193, and Thr226. The hydrophobic residues of the BACE-1 are in contact with the 3NCP are Leu36, Ser41, Thr78, Phe114, Ile124, Asp223, and Gly225. The binding mode and chemical interactions profile of the 3NCP are given in Figure 1.

#### 2.1.2. Molecular Dynamics (MD) Simulations

**Figure 2:**
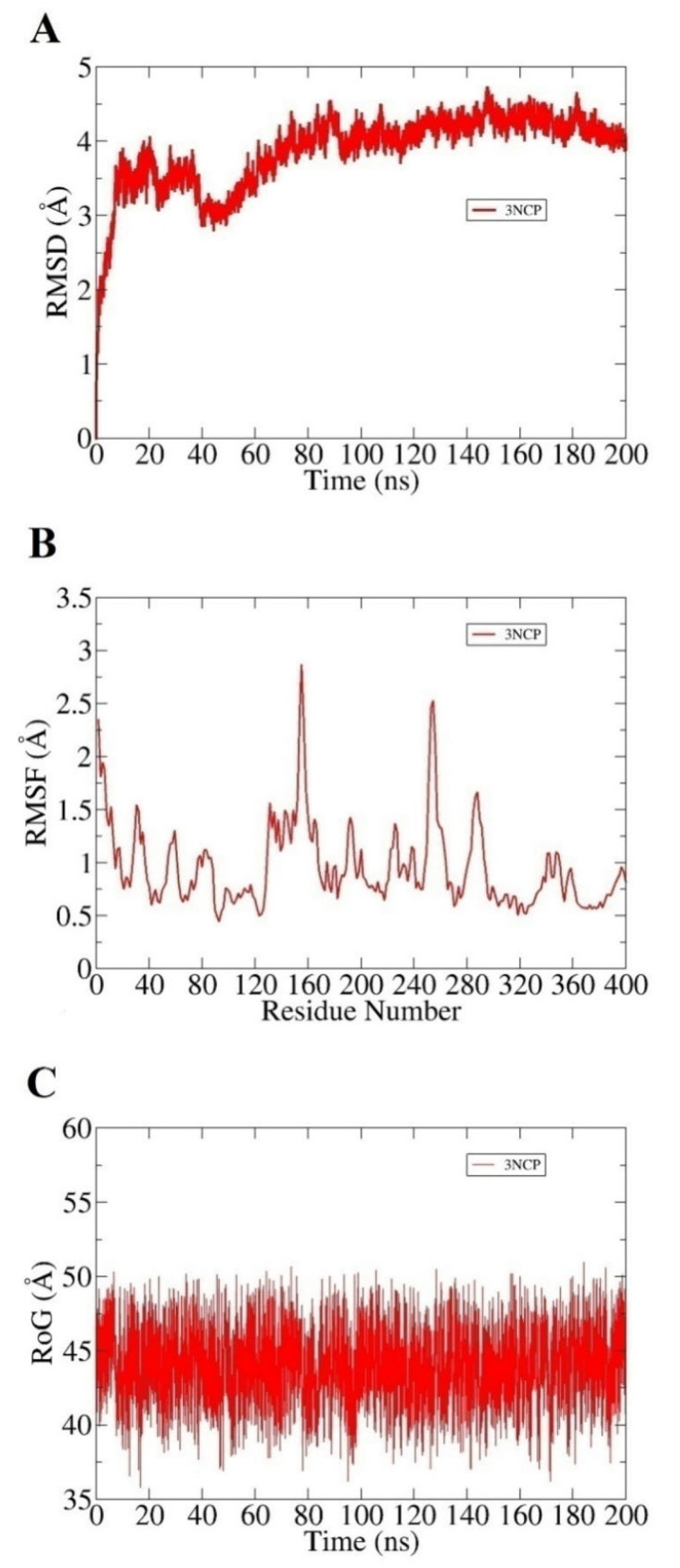
System stability evaluation based on the MD simulation trajectories. (**A**) RMSD, (**B**) RMSF, and (**C**) RoG.

Both complexes were subjected to 200 ns of MD simulation to investigate the systems’ dynamics and infer intermolecular stability. The complexes were observed in good structural stability throughout the simulation time though some initial dynamics adjustments and can be noticed in the first 10 ns. Root mean square deviations (RMSD) plot for the system is provided in Figure 2A. The average RMSD of the 3NCP- BACE1 complex is 1.93 Å. This suggests that the compound-bound state of the BACE1 gained significant stability as the simulation proceeds. This further validates that after some initial adjustments of the compound conformation at the docked cavity, the compound binding mode predicted by the docking remained highly stable and showing a high affinity for the enzyme pocket residues. RMSF analysis was additionally carried out to confirm the stability of enzyme residues in the presence of the compound (Figure 2B). Fluctuations of the enzyme residues were plotted against time to decipher the regions of the enzyme that were more stable as well as to point out unstable regions of the enzyme. Overall, the mean RMSF of the system is 2.8 Å. Upon complex visualization, it was indicated the initial binding conformation of the compound was changed a bit to dock deep inside the pocket and get significant binding with functional pocket residues. This resulted in putting pressure on flexible regions of the enzyme such as loops to properly hold the compound at the active site. The radius of gyration (RoG) analysis was performed to examine compact packing of the BACE1 secondary structural elements in the presence of the enzyme (Figure 2C). It can be noticed in Figure 2 that the enzyme is highly compact and does not show any structural variations in the presence of the compound. This demonstrates that the enzyme-compound systems are highly stable, and the compound is enjoying its stay at the active pocket of the enzyme. The mean RoG value of the 3NCP is 43.8 Å. 

#### 2.1.3. Molecular Mechanics-Generalized Born Surface Area (MMGBSA) Binding Free Energy Analysis 

The MMGBSA method of binding free energy has been widely applied in studying receptor-ligand interactions and is more reliable than conventional docking studies. It was revealed that the system is highly stable as predicted by docking and simulation studies. The net binding energy of the system is <−12 kcal/mol, demonstrating the high equilibrium nature of the complexes. The van der Waals energy, as well as the electrostatic energy, contributed significantly to the net binding energy of the systems. However, the polar energy of the systems contributed unfavorably to the overall solvation energy in contrast to non-polar energy. A detail about the contribution of each energy term to the net binding energy is given in Table 1.

### 2.2. Behavioral Experiments

#### 2.2.1. Effect of 3NCP in the LD Test

**Figure 3:**
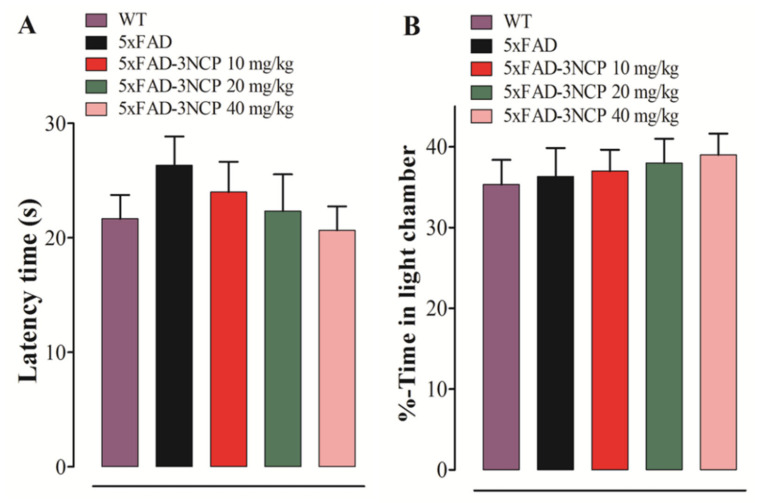
Effect of the 3NCP on time latency to enter the dark box from light box (**A**) and the percent time spent in the light chamber (**B**) in the light/dark box test.

The latency time of the mouse to enter from the light into the dark box and the time spent in the light compartment were evaluated. No difference in the latency and time spent in the light compartment was observed between all groups (Figure 3).

**Figure 4:**
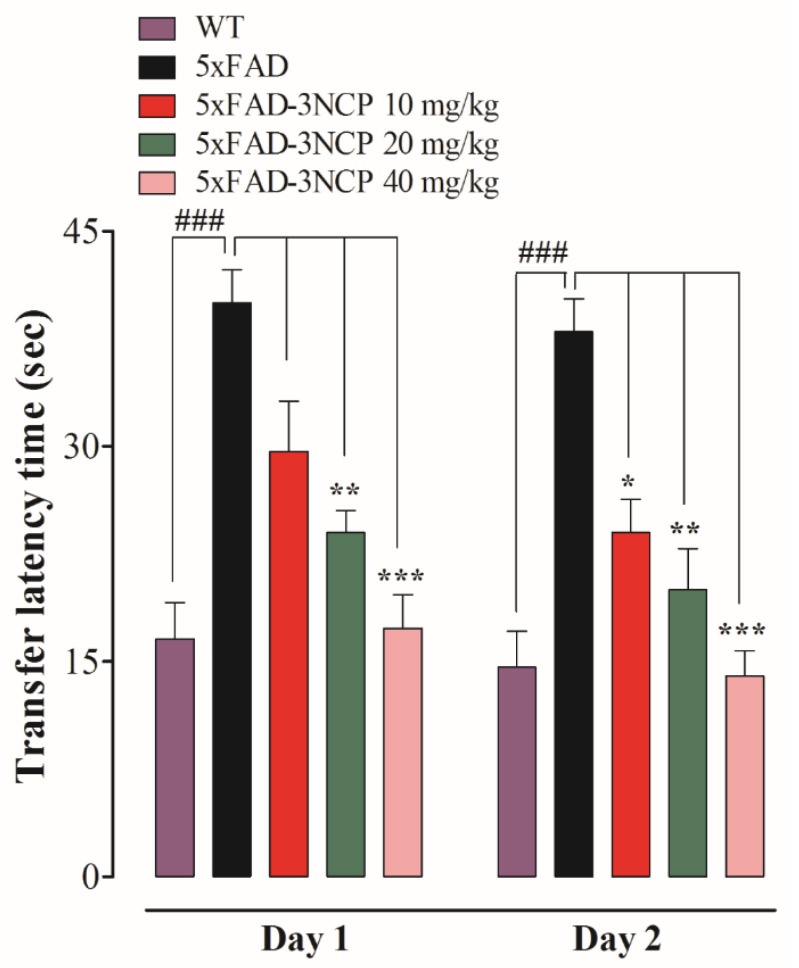
Effect of the 3NCP on the memory of 5xFAD mice in the EPM test. Data were analyzed with one-way ANOVA followed by the Dunnett post hoc test. * *p* < 0.05, ** *p* < 0.01, *** *p* < 0.001 compared to the 5xFAD-group and ### *p* < 0.001 compared to the WT group.

The effect of 3NCP on learning, memory and the responses to novel situations were assessed by observing their exploration of two different environments, relatively. The transfer latencies (TL) in acquisition (day 1) and retention (day 2) trials were noted for all mice. The 5xFAD mice exhibited more transfer latency time in acquisition and retention trials in comparison with wild type mice (*p* < 0.001), whereas the 5xFAD mice treated with the 3NCP (10 mg/kg) have taken less time in transfer from the open-arm into the close-arm as compared to the 5xFAD mice in the retention trial (*p* < 0.05). The mice treated with the 3NCP (20 mg/kg) exhibited a decrease in the TL in both trials as compared to the 5xFAD mice treated with the vehicle (*p* < 0.01, *p* < 0.001). The 3NCP (40 mg/kg) treatment showed a more significant reduction in transfer latency time in both trials (*p* < 0.001) (Figure 4).

#### 2.2.2. Effect of 3NCP in the Barnes Maze Test

**Figure 5:**
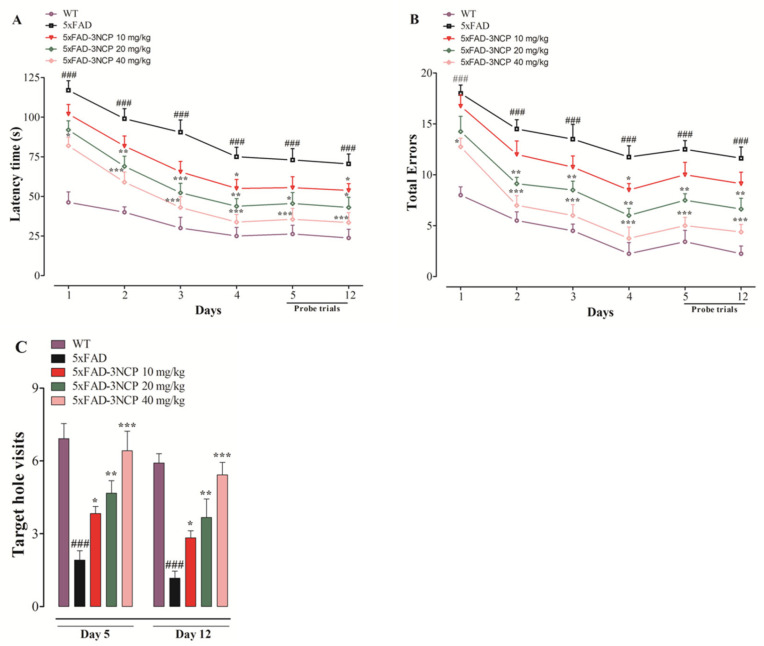
The latency time to search target hole (**A**), and total errors (**B**) of 3NCP treated mice during training and probe trials, and the target hole visits (**C**) during the probe trials in the Barnes maze test. Bars express mean ± SEM. * *p* < 0.05, ** *p* < 0.01, *** *p* < 0.001 compared to the 5xFAD mice, ### *p* < 0.001 compared to the non-transgenic WT mice. Data were analyzed through two-way ANOVA, followed by Bonferroni and one-way ANOVA, followed by Dennett post hoc tests.

The latency time to reach the target-hole, and total errors in finding the target-hole, was observed in the Barnes maze test during training and probe trials. The number of target-hole visits during probe trials was also observed. The 5xFAD mice exhibited a significant increase in latency during training and probe trials compared to the WT mice (*p* < 0.001). The 3NCP (10 mg/kg) shows a significant decrease (*p* < 0.05) on days 4 and 12 at a20 mg/kg dose, caused significant decline (*p* < 0.05, *p* < 0.01, *p* < 0.001) on days 2, 3, 4, 5, and 12, while at 40 mg/kg dose, and indicated a significant decrease (*p* < 0.05, *p* < 0.001) on days 1, 2, 3, 4, 5, and 12 in the latency time in finding the target hole. The 3NCP (10 mg/kg) depicted a significant decline in total errors on day 4 (*p* < 0.05), at 20 mg/kg dose exhibited a significant decline in total errors on days 2, 3, 4, 5, and 12 (*p* < 0.01), and at 40 mg/kg dose exhibited a significant decrease in total errors on days 2, 3, 4, 5, and 12 (*p* < 0.001) during training and probe trials. The 3NCP (10 mg/kg) caused an increase (*p* < 0.05) on days 5 and 12, at 20 mg/kg exhibited a significant increase (*p* < 0.01) on days 5 and 12, and at 40 mg/kg dose depicted a significant increase (*p* < 0.001) on days 5 and 12 in the number of target hole visits (Figure 5).

### 2.3. Effects of 3NCP on Aβ Plaques in Frontal Cortex and Hippocampus 

**Figure 6:**
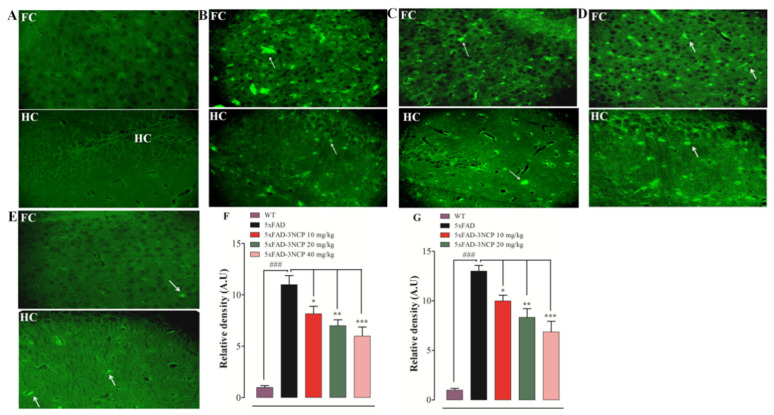
The protective effects of 3NCP on Aβ plaques in the HC and FC of the 5xFAD mice model. FC and HC regions of the wild type of non-transgenic mice treated with vehicle (**A**), FC and HC regions of the transgenic 5xFAD mice treated with vehicle (**B**), FC and HC regions of the 3NCP (10, 20, 40 mg/kg) treated 5xFAD mice (**C**–**E**), and the relative density of Aβ plaques in FC (**F**) and HC (**G**). The data were analyzed through one-way ANOVA followed by the Dunnett post hoc test. * *p* < 0.05, ** *p* < 0.01, *** *p* < 0.01 compared to the 5xFAD-mice, ### *p* < 0.001 as compared to WT-mice.

**Figure 7:**
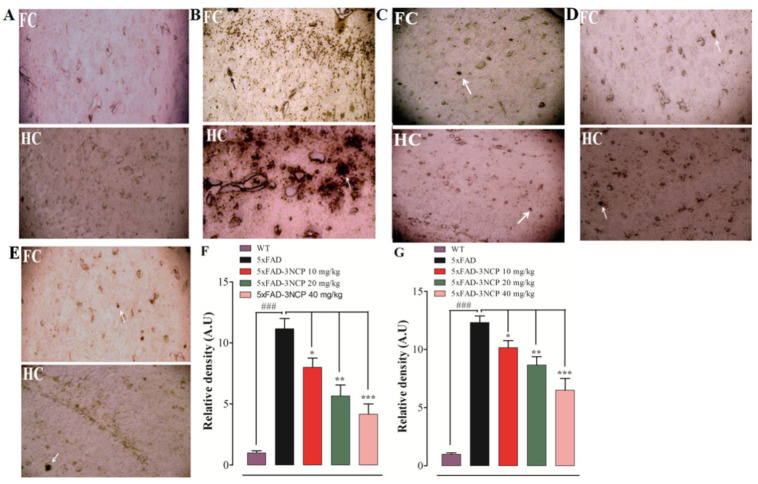
The protective effects of 3NCP on Aβ plaques in the HC and FC of the 5xFAD mice model. Neuritic plaques were detected using an anti-Aβ antibody 4G8 and were developed using the ABC and DAB methods. FC and HC regions of; non transgenic wild type mice treated with vehicle (**A**), transgenic 5xFAD mice treated with vehicle (**B**), and 3NCP (10, 20, 40 mg/kg) treated 5xFAD mice (**C**–**E**), and the relative density of Aβ plaques in FC (**F**) and HC (**G**). The data were analyzed by one-way ANOVA followed by a post hoc Tukey’s analysis. * *p* < 0.05, ** *p* < 0.01, *** *p* < 0.01 compared to the 5xFAD-mice, ### *p* < 0.001 as compared to WT-mice.

The Aβ plaques are another pathological hallmark of the AD; the level of these plaques in the FC and HC were evaluated. The findings of the histopathological study indicated that the 5xFAD mice showed vigorous and clear Aβ plaques in the FC and HC in comparison with the WT group (*p* < 0.001). However, the 5xFAD mice treated with the 3NCP (10–40 mg/kg) displayed a significant decline in the Aβ plaques load in the FC and HC regions in comparison with the 5xFAD mice (*p* < 0.05, *p* < 0.01, *p* < 0.001) (Figure 6 and Figure 7). 

### 2.4. Effect of 3NCP on BACE-1 Expression 

**Figure 8:**
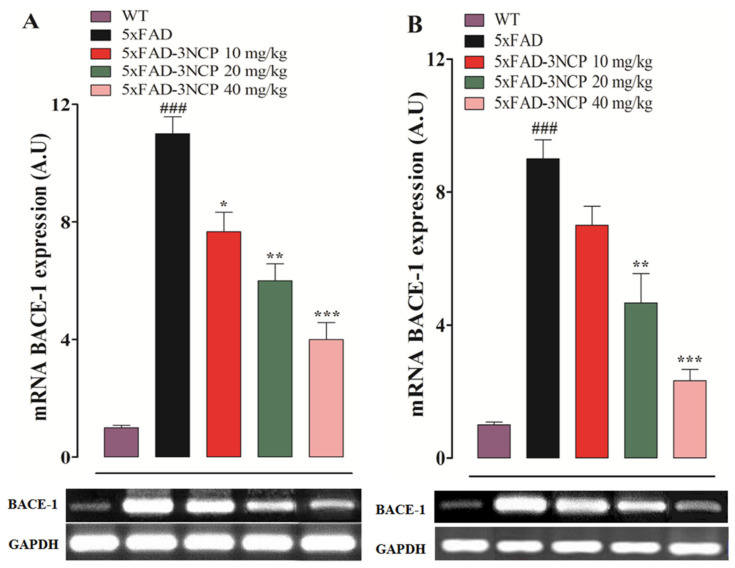
Effect of the 3NCP on the mRNA expression of BACE-1 in the hippocampus (**A**) and frontal cortex (**B**) was measured. The results were obtained using ImageJ software and expressed in an arbitrary unit (A.U). * *p* < 0.05, ** *p* < 0.01, *** *p* < 0.001 compared to the 5xFAD-mice and ### *p* < 0.001 compared to the WT-mice.

The effect of 3NCP on BACE-1 enzyme expression in the hippocampus and frontal cortex was investigated through RT-PCR. The transgenic 5xFAD mice treated with the vehicle showed increased expression of the enzyme in both areas (*p* *<* 0.001), while treatment with 3NCP significantly reduced its expression in both tissues (*p* *<* 0.05, *p* *<* 0.01, *p* *<* 0.001) (Figure 8).

## 3. Discussion

In AD, brain cells that process, store and retrieve information degenerate and die. There are many culprits involved in this destruction. One of these culprits is microscopic brain protein-fragments known as beta-amyloid that accumulate in the brain, disrupting communication between brain cells [30].

The light/dark box, elevated plus maze (EPM), and Barnes maze tests were conducted for determining the effect of the 3NCP on the behavior of the test animals in terms of anxiety and spatial learning and memory, respectively [31,32,33]. The light/dark box test is used for the assessment of anxiety and locomotion. The latency time to enter the dark compartment and the time spent in the light compartment were recorded from the light/dark test. Mice tested in the light/dark box resulted in showing no significant difference in time latency and time spent in the light compartment among all groups of animals, implying that the 3NCP did not cause any anxiety.

The EPM test is used for the assessment of anxiety, spatial learning and memory in mice [34,35]. Reduce latency time (TL) in the EPM task indicated the improvement in memory and learning, while increase TL indicates memory impairment [36]. The results of the EPM test showed that the 3NCP reduced TL in the retention phase indicates improved memory.

The Barnes maze was first introduced by Carol Barnes to minimize the swimming-induced stress of rats in the Morris Water Maze (MWM), and then it was adapted to mice. Animals in this test were placed at the center of the BM apparatus and receive negative reinforcement (loud buzzing, bright light, etc.) to motivate them to escape through a target hole to the box. It is also used for the investigation of spatial reference memory and learning without inducing anxiety and fear that are common in MWM [37]. The 5xFAD mice exhibited deficits in spatial memory and learning in this model, whereas the 3NCP treated mice showed improvement in the spatial memory and learning.

Amyloid-beta plaques are key components of Alzheimer-associated pathologies, including aberrant synaptic plasticity, inflammation, oxidative toxicity, and memory impairment [38,39]. In AD patients, pathological alterations occur in the brain shape, including the formation of Amyloid plaques. Amyloid plaques develop in the hippocampus and cortical area of the brain due to the increased production of Amyloid beta peptides [40]. The elevation of Amyloid-beta in the brain is linked with cognitive impairment [41]. Prevention of these plaques is important for the treatment of AD. That’s why the effect of the 3NCP on Amyloid-beta plaques was tested through thioflavin-S staining and immunohistochemistry techniques. The findings showed that the 3NCP (10–40 mg/kg) significantly reduced the formation of the plaques in the hippocampal and cortical tissues as compared to the 5xFAD mice.

β-Secretase is a membrane-bound aspartyl protease enzyme that cleaves APP and its other substrates to the exterior of the bilayer [42]. There are two major types of the β-Secretase, BACE-1 and BACE-2 having 64% amino acid similarities [43]. BACE-1 is highly expressed in the brain and mainly responsible for Aβ production, while the level of BACE2 is very low in the brain [44]. It has been reported that the BACE1 is the major β-secretase activity in the brain [45]. The BACE-1 activity and Aβ protein concentrations are increased in the AD brain [46]. The results of the computational study revealed that the 3NCP has the potential for the beta-site APP cleaving enzyme-1(BACE-1) inhibition, which is further confirmed by the RT-PCR study, and showed that the 3NCP significantly reduced the BACE-1 expression in the brain leading to the decreased production of beta-amyloid plaques.

The 3NCP alleviates memory impairment, reduced plaque formation, and inhibits BACE-1 activity, displaying activities comparable or higher than the reported drugs such as melatonin, quercetin, and rutin [47,48,49].

Findings of this study showed the beneficial effects of the 3NCP in the 5xFAD mice model of Alzheimer’s disease. This study also has some limitations. We did not evaluate the effect of the 3NCP on α and γ-secretase, BACE-2, overall gliosis, neurons degeneration, APP level, and tau protein. Cytotoxicity was not observed at the concentration used in this study. The most beneficial effects of the 3NCP were detected at the highest dose of 40 mg/kg. Furthermore, improved cognitive performance with essential changes in the behavioral performance was noted, whereas a decrease in beta-Amyloid formation was found to have a potential association in the amelioration of the above parameters in the 5xFAD mice. Moreover, the exploration of the relationship between beta-Amyloid plaques formation and AD risk may help us in understanding AD pathogenesis and to develop better treatment strategies.

## 4. Materials and Methods

### 4.1. Materials

Agarose, dimethyl sulfoxide, ethidium bromide, hydrogen peroxide, paraformaldehyde, sucrose, Thioflavin-S- stain, Tris EDTA (Sigma Aldrich, St. Louis, MO, USA), biotin labeled 4G8 antibody (BioLegend, San Diego, CA, USA), boric acid (Serva, Heidelberg, Germany), DNA extraction kit (BioShop® Canada, Burlington, ON, Canada), cDNA synthesis kit (ABM, Vancouver, BC, Canada), DNA ladders (Invitrogen, Carlsbad, CA, USA), entellan (Electron Microscopy Sciences, Hatfield, PA, USA), ethanol, diethyl ether (Merck, Darmstadt, Germany), formaldehyde (BDH Chemicals Ltd, Poole, UK), 2X PCR master mix, Taq polymerase, skim milk, PCR grade water, formic acid (Thermo Fisher Scientific, Waltham, MA, USA), Imm PACT DAB, Elite ABC kit (Vector Laboratories, Burlingame, CA, USA), paraffin wax (Bio Optica Milano, Spa, Milan, Italy), PCR primers (Macrogen, Seoul, Korea), Superfrost* Plus microscope slides (Dako, Agilent, Santa Clara, CA, USA), TRI-reagent (BioShop® Canada, Burlington, ON, Canada), tris, tween 80 (Scharlau, Barcelona, Spain), and triton X-100 (Duksan, Kyungkido, Korea), and xylene (Lab Scan Ltd, Dublin, Ireland) were used in this study. 

### 4.2. Animals

The 5xFAD mouse model of Alzheimer’s disease was used in this study and these mice were obtained from the Jackson Laboratory US. The 5xFAD mice are useful models for studying human FAD because the brains of these mice demonstrate an increase Aβ42/Aβ40 ratio, axonal degeneration, impairment in motor functions, and working memory deficits, as observed in human FAD [50,51]. The 5xFAD mice of either sex (age: 5–6 months, weight: 28–33g) were used as a disease group and non-transgenic wild-type mice littermates were used as a control group (*n* = 10/group). Animals were bred and kept at 22 ± 2 °C with a 12/12 h light/dark cycle in the animal house at the Department of Pharmacy, University of Peshawar, Pakistan. In vivo experiments were carried out following Animals’ Scientific Procedures Act (UK), 1986, and procedures were approved by the ethical committee of the Pharmacy Department, University of Peshawar (reference number 12/EC-17/Pharm).

### 4.3. Computational Study of 3NCP with β-Secretase

#### 4.3.1. Molecular Docking with AutoDock 4.2

The crystal structure of the BACE1 enzyme was fetched from the protein data bank (PDB) available under PDB id: 6OD6 [52], resolution of 2 Å and R-value of 0.22 in UCSF Chimera and subsequently subjected to receptor preparatory phase [53]. First, associated co-crystallized ligands and water molecules not relevant for enzyme functionality were discarded and then the enzyme was saved in pdb format. Afterward, polar hydrogen atoms were added, and the appropriate number of Gasteiger charges was assigned to the enzyme structure in the AutoDock tool [54,55]. The grid box was set covering both catalytic aspartate (Asp) residues (Asp38 and Asp223) [56]. Both the mentioned residues are critical in enzyme functionality and their inhibition leads to the blocking of the enzyme activity. The grid box was centered at 24.693 Å (X-axis), 95.985 Å (Y-axis) and 6.459 Å (Z-axis) which give the grid total size on XYZ as 60 Å. All the mentioned information about the receptor, ligands, and grid center, and size were recorded in the configuration (.conf) file. Binding modes generated at the docked site for ligand during the docking run were allowed to 100. The docking protocol was validated first by re-docking the co-crystallized M7D compound blindly to the BACE1 that revealed an RMSD of 1.02 Å, thus the docking protocol is suitable for docking of our desired compounds [57]. The binding affinity of the compounds to the enzyme was measured in terms of binding energy; a lower binding energy implied a high affinity of binding of the compounds to the receptor enzyme. The top-ranked binding conformation of the compound was complexed with the enzyme and saved in.pdb format for additional visualization and atomic level chemical interactions.

#### 4.3.2. Molecular Dynamics Simulation 

The selected complex was simulated for 200 ns employing Amber20 [58] per the steps followed by Asma et al., 2018, with a slight modification [59]. The enzyme and compound parameters were defined using FF14SB [60] and a GAFF force field [61], respectively, and their topologies were recorded via the LEAP module [62]. The system was then placed in a TIP3P solvation box with a padding distance of 12 Å. Energy minimization of the system was performed at 1500 rounds of the steepest descent and gradient algorithms were conjugated. The complex was then headed for 50 ps in a gradual fashion of temperature increase from 0 to 300 K, and subsequently equilibrated for 1 ns. The production run of MD simulation was performed along 200 ns in presence of isothermal-isobaric ensemble, pressure, and temperature (NPT), 1 atm pressure, Langevin Thermostat (ntt = 3), and barostat of Monte Carlo. The MD simulation trajectories were analyzed in terms of RMSD, RMSF (root mean square fluctuations), and RoG using the CPPTRAJ module [63].

#### 4.3.3. Binding Free Energy Calculations

Validation of the docking and simulation experiments were validated one step further by estimating binding free energy of the complex using the MM/GBSA method [64]. MM/GBSA calculations were carried out for the estimation of the relative binding affinity of ligands to receptors and to understand the binding mode of molecules to receptors [65].

### 4.4. Genotyping of 5xFAD Mice

Methodology of genotyping as specified by Jackson Lab U.S. for strain transgenic mice was followed with slight modifications [23]. DNA extraction was carried out by using a DNA extraction kit following the manufacturer’s protocol. The main steps of that protocol were tissue cutting and grinding, homogenization, cell lysis, DNA binding by adding ethanol, washing of GS column, DNA elusion, and quantification. After DNA extraction the PCR amplification occurred, and the internal positive control (F- 5′-CTAGGCCACAGA-ATTGAAAGATCT-3′, R- 5′-GTAGGTGGAAATTCTAGCATCATCC-3′) and APP transgene (R- 5′-AGGACTGACCACTCGACCAG-3′, R- 5′-CGGGGGTCTAGTTCT-GCAT-3′) primers were used in the genotyping process. Amplification of PCR was confirmed by running a mixture of PCR products (5 μL) and 6× loading dye (1 μL) in each well of 1 to 2% agarose gel in 1× TBE buffer for 30 min at a constant voltage of 80 V. A solution of 5 μL of ethidium bromide (1%) was added to 100 mL gel solution [66]. The amplified products were visualized under UV light using the transilluminator (Life Technology, Carlsbad, CA, USA).

### 4.5. Behavioral Experiments

Animals were treated for 28 days; drugs were administered intraperitoneally (i.p.) once a day. The behavioral activities were carried out from 9:00 a.m. to 5:00 p.m. The light/dark box test was performed on day 14 and elevated plus-maze on days 15 and 16, while the Barnes maze test was conducted on days 17–28, respectively. The animals were sacrificed on day 28 for further studies.

#### 4.5.1. Light/Dark Box (LD) Test

The mice’s tendency to explore differentially the light and dark regions was measured. The light/dark box was constructed in our lab and consisted of two compartments: light and dark. Dimensions of the compartment were 1/3 for the dark and 2/3 for the light, with an exterior size of 46:27:30 cm (lbh). The opening between the two compartments is 7 cm. Every animal was placed in the light compartment for five min and the time latency to enter the dark, and the percent time spent in each compartment were calculated [67,68].

#### 4.5.2. Elevated Plus Maze (EPM) Test

The EPM test is used for testing anxiety as well as for learning and memory. The EPM apparatus contains two opposite open- and two opposite close-arms of the same size (length = 30 cm, width = 6 cm), has high walls (15 cm), and is elevated 40 cm off the floor. On the first day, each mouse facing away from the center was positioned at the end of the open-arm. The time in which the mouse moves from the open- into the close-arm with all legs is called transfer latency (TL) and was noted for each mouse on day one. If the mouse did not enter the close-arm within 1.5 min, it was pushed gently into the close-arm and TL was assigned as 90 s. Mice were allowed no more than 120 s to explore the EPM apparatus. Retention of this task (memory) was evaluated on the next day [69].

#### 4.5.3. Barnes Maze (BM) Test

This test was used for testing memory and spatial learning. The Barnes maze was constructed in our lab from wood and consisted of a circular platform (92 cm in diameter) with twenty holes, having one hidden escape box. This test consisted of three trials: habituation, training, and probe. The habituation trial is a one-day trial performed to habituate the animal to the maze by placing it at the center of the maze in a chamber, removing the chamber after 10 s, and starting the buzzer. The animals were guided to the targeted escape box and once they enter to box individually, the buzzer is switched off. After the habituation trial, training trials were carried out for four days; animals were given four trials per day with 15 min time intervals between the two trials. In training trials, the animal in the starting chamber was placed at the center of the maze. After 10 s, the chamber was removed, allowing the animal to explore it for three mins. The number of errors and latency to reach the escape hole was recorded. After training trials, the probe tests were conducted on days 5 and 12 to ascertain if it remembers the location of the escape box. The target-hole was closed, and mice were allowed 90 s to explore the maze. Time spent to reach the target and the number of errors (nose pokes in other holes) were recorded [70].

### 4.6. Detection of Neuritic Plaques in Mouse Brain

#### 4.6.1. Thioflavin S Staining Procedure for Detection of Neuritic Plaques

Following the brain extraction and fixation, hemi brain sections were mounted on a glass slide and air-dried before staining. The slides were washed with ethanol, incubated in the thioflavin-S solution for 15 mins, and followed by a washing with ethanol and distilled water. The slides were mounted in aqueous media, dried in dark for 2 h, and protected with coverslips. The fluorescent microscope (Olympus, Tokyo, Japan) was employed to visualize any plaques [71].

#### 4.6.2. Immunohistochemistry (IHC)

Mice were sacrificed by decapitation under ether anesthesia and brains were extracted. Sectioning and embedding of the brain were carried out after brain extraction. Each section was removed on a plate and washed with Tx-PBS (Phosphate buffer saline). Then, each section was blocked with non-fat skim milk (5%), incubated for 12 h in the primary antibody at 4 °C, and washed with Tx-PBS. Each section was then incubated in an already prepared ABC solution for 30 min and washed with Tx-PBS. The section was incubated for 5 to 10 min in 3, 3-diaminobenzidine tetrahydrochloride (DAB) mixture and washed with Tx-PBS. Then, brain sections (free-floating) were mounted on gelatin-coated slides, air dried, cleared with xylene, and mounted in the entellan. The plaques were visualized through a microscope [71].

### 4.7. RT-PCR

The total RNA was extracted from hippocampal and frontal cortex tissues using TRI-reagent. The total RNA was converted into cDNA through a cDNA synthesis kit. The expression of BACE-1 was determined. The primers used in this study were BACE-1 Forward: 5′-ACCTGGTGAGCATCCCTCAT-3′ Reverse: 5′-CCTCCCAGTTGG-AACCATTG-3′, and GAPDH Forward: 5′-TGCACCACCAACTGCTTAGC-3′, Reverse: 5′-GGCATGGACTGTGGTCATGAG-3′. The amplification products were separated via 1.5% agarose gel and visualized through a UV transilluminator [23,72].

### 4.8. Statistical Analysis

The BM tests data were analyzed through two-way ANOVA followed by a Bonferroni test, while the data of LD box, EPM, and RT-PCR studies were analyzed by one-way ANOVA followed by the Dunnett posthoc test. Analyses were performed through GraphPad Prism 5, statistical significance was set at *p* < 0.05 and the results were expressed as mean ± S.E.M.

## Figures and Tables

**Table 1:** MM/GBSA Derived Binding Free Energies of 3NCP-BACE1 Complex Estimated Using Different GB Models.

Table Cont.	3NCP Complex
Van der Waals energy	−27.72
Electrostatic energy	−37.01
Polar solvation energy	47.36
Nonpolar solvation energy	−3.6
Net gas phase energy	−64.73
Net solvation energy	43.76
Net total energy	−20.97

## Data Availability

The authors declare that the data supporting the findings of this study are available within the article.
